# Structured LDH/Bentonite Composites for Chromium Removal and Recovery from Aqueous Solutions

**DOI:** 10.3390/molecules28124879

**Published:** 2023-06-20

**Authors:** Mitra De Geest, Bart Michielsen, Radu-G. Ciocarlan, Pegie Cool, Elena M. Seftel

**Affiliations:** 1 Laboratory of Adsorption & Catalysis, University of Antwerp, Universiteitsplein 1, 2610 Wilrijk, Belgium; 2 VITO Flemish Institute for Technological Research, Boeretang 200, 2400 Mol, Belgium

**Keywords:** layered double hydroxide, bentonite, clay composites, chromium abatement, chromium recovery

## Abstract

This study focuses on chromium removal through adsorption and ion exchange using structured calcined layered double hydroxide (LDH) (MgAl)–bentonite composites. Firstly, the powders were structured into granulates to study the effect on Cr sorption kinetics to circumvent the limitations of working with powders in real-life applications. Secondly, the regeneration of the structured composites was optimized to enable multi-cycling operation, which is the key for their applicability beyond laboratory scale. Firstly, the LDH/bentonite ratio was optimized to obtain the best performance for the removal of Cr^3+^ and Cr^6+^ species. In powder form, the calcined adsorbent containing 80 wt% LDH and 20 wt% bentonite performed best with an adsorption capacity of 48 and 40 mg/g for Cr^3+^ and Cr^6+^, respectively. The desorption was optimized by studying the effect of the NaCl concentration and pH, with a 2 M NaCl solution without pH modification being optimal. The kinetic data of the adsorption and desorption steps were modelled, revealing a pseudo-second order model for both. This was also demonstrated using XRD and Raman measurements after the Cr^3+^ and Cr^6+^ adsorption tests, indicating successful uptake and revealing the adsorption mechanism. Finally, five consecutive adsorption–desorption cycles were performed, each showing nearly 100% adsorption and desorption.

## 1. Introduction

By 2025, approximately four billion people will reside in areas with scarce water resources, while water demand will increase annually [1,2]. Water pollution is a major contributor and results from industry, acid rain, dumping of human waste and sanitation [3]. As a result of these pollution sources, toxic metals, organic molecules, dyes and drugs end up in the environment [4,5,6,7,8]. Chromium-containing compounds contribute to this water pollution, as it comes into the wastewater streams from multiple industries such as dyeing, leather tanning and metallurgy [9]. Consequently, a solution for the removal of toxic chromium compounds—which are also a part of carcinogenic, mutagenic and/or reprotoxic materials (CRMs)—is needed. Moreover, to reduce the expenses for chromium-based processes, its recovery is an essential criterion for prospective techniques.

Consequently, adsorption is a promising technique, since it is highly flexible in design and operation and allows the reuse of the adsorbents through desorption processes [10]. Therefore, a variety of adsorbents have been investigated in the literature, of which clay materials are amongst the most suitable adsorbents due to their eco-friendliness, adsorption and ion exchange capacity, water dispersibility, low cost and availability [11,12,13,14]. Clays can be subdivided in anionic (i.e., layered double hydroxides (LDHs)) and cationic clays, being characterized by a positive and negative layer charge, respectively. Consequently, a composite material of anionic and cationic clays provides the opportunity to target both abundant forms of chromium (i.e., Cr^3+^ and Cr^6+^ as CrO_4_^2−^ oxyanions). Unfortunately, the µm to sub-µm sizes of the clays bring forth potential problems for the release of the used adsorbents into the environment, which can be tackled through shaping or deposition onto a template, with the synthesis of Mg-Al metal oxides on filter paper being an example of the latter [15,16]. Moreover, it is possible to implement magnetic properties into the adsorbents, which will also facilitate its removal after usage, but this is less practical in real-life applications due to the requirement of an external magnetic field [17]. This may be regarded as a drawback for its implementation; however, this can be circumvented by shaping the adsorbents into granulates. Moreover, granulates can be integrated in filter units or columns, enabling their applicability in real environmental processes, such as depollution of chromium-containing wastewaters. Consequently, by implementing this feature into the prepared adsorbents, an advantage is obtained compared to the state-of-the-art.

Examples of state-of-the-art LDH, bentonite and LDH/bentonite adsorbents used for chromium removal are provided in Table 1. Bentonite, a phyllosilicate, is an example of a cationic clay and is an off-white colored assembly of clay minerals, resulting from the weathering of volcanic ash or the alteration of silica-bearing rocks [18,19]. The material predominantly consists of montmorillonite and additionally contains kaolinite, illite, cristobalite, carbonates, zeolites, iron oxides and hydroxides, quartz and feldspar [20,21]. LDH materials, on the other hand, are anionic-type clays and are characterized by a layered clay-type structure having [M_1−x_^II^M_x_^III^(OH)_2_]^x+^[A^−q^_x/q_.n H_2_O]^x−^ as their chemical formula. The layers are composed of divalent (M^II^) and trivalent (M^III^) cations, while the interlayer galleries contain charge-neutralizing anions (A^−q^) and water. Subsequently, layered double oxides (LDOs) can be obtained by thermal treatment of the corresponding LDHs. However, almost all provided examples (authors of [22,23] used immobilized films and membranes, respectively) concern powders and not shaped adsorbents, which makes them less useful for real-life applications as explained above. Furthermore, the influence of shaping on the adsorption (and desorption) is not evaluated. In contrast, the prepared adsorbents are shaped into a practical form, making them more applicable for real-life applications. 

Moreover, when evaluating the state-of-the-art on Cr^6+^ and Cr^3+^ sorption, it can be noted that the focus is mostly on the adsorption. Namely, desorption studies can be found in the state-of-the-art; however, these are very limited [37,42,43]. Hence, efforts were made to optimize desorption, thus enabling the regeneration of the adsorbent and, consequently, the ability to perform multiple cycles.

In summary, the objective of this study was the removal of both Cr^6+^ and Cr^3+^ ions from aqueous solutions. In this regard, composites of a Mg-Al LDH and bentonite were prepared and shaped into granulates, with the shaping enabling the limitation of the unintentional environmental release of the adsorbent and, moreover, the possibility to pack the adsorbent in a column. The obtained granulates were calcined to achieve the final mechanical strength of the granulates, and consequently Mg-Al LDO/bentonite composites were obtained. The Mg-Al LDH/bentonite ratio was varied to optimize the adsorption capacity while their material properties and Cr sorption performance were evaluated. Finally, the desorption and five adsorption–desorption cycles were performed to evaluate the adsorbent’s recyclability. The desorption experiments with a removal efficiency up to 100% clearly indicate the possibility to recover Cr^6+^- and Cr^3+^-based compounds for reuse, which is beneficial for real-life applications.

## 2. Results and Discussion

### 2.1. Structural Characterization

**Figure 1:**
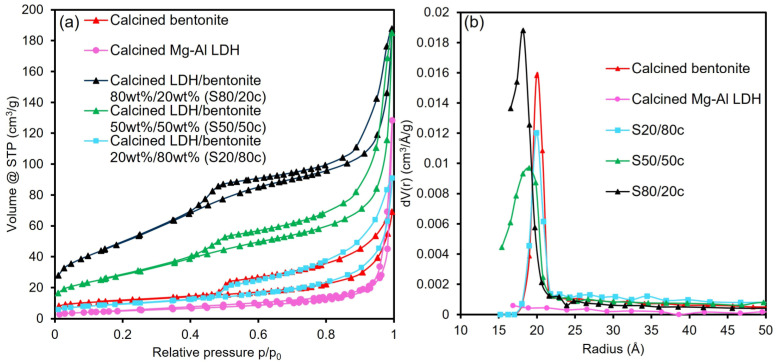
(**a**) N_2_ sorption isotherms; (**b**) pore size distributions.

The N_2_ sorption isotherms, pore size distributions and physicochemical properties of the samples are displayed in Figure 1a,b and Table 2, respectively. Figure 1a reveals a type IV(a) isotherm for all samples, which is indicative of a mesoporous material. Moreover, the calcined bentonite and the calcined Mg-Al LDH display a H4 and H3 type hysteresis loop, respectively. A H4 hysteresis loop is characteristic of slit-shaped pores, while a H3 type hysteresis loop suggests the presence of either slit-shaped pores or aggregates of flat particles. The latter hysteresis type can be obtained for nonrigid aggregates of plate-like particles such as clays. Based on the comparison between the calcined starting materials and composites, it can be concluded that the adsorbents resemble both the calcined Mg-Al LDH and bentonite. However, the higher the bentonite fraction, the more the hysteresis loop resembles the calcined bentonite. In this regard, it can be observed that both the calcined LDH/bentonite 80 wt%/20 wt% (S80/20c) and LDH/bentonite 50 wt%/50 wt% (S50/50c) samples have a H3 type hysteresis loop, while the LDH/bentonite 20 wt%/80 wt% (S20/80c) adsorbent displays a type H4 hysteresis loop. Additionally, it can be observed from Table 2 that both the pore volume and the BET specific surface area (S_BET_) decrease with the increasing amount of bentonite. Furthermore, it can be observed from Figure 1b that a shift occurs in the pore size distribution when the bentonite fraction increases, which explains the decrease in the total pore volume. Namely, fewer pores can be present due to the increase in pore size, and this results in the perceived decrease. This affects the available surface and, consequently, the BET specific surface area. Moreover, Hg intrusion was performed on the two size fractions of the S80/20c sample, with the results being included in Appendix A (Table A1). As can be seen from Table A1, a difference in granulate size negligibly influences the total cumulative volume, average pore radius and total porosity.

**Figure 2:**
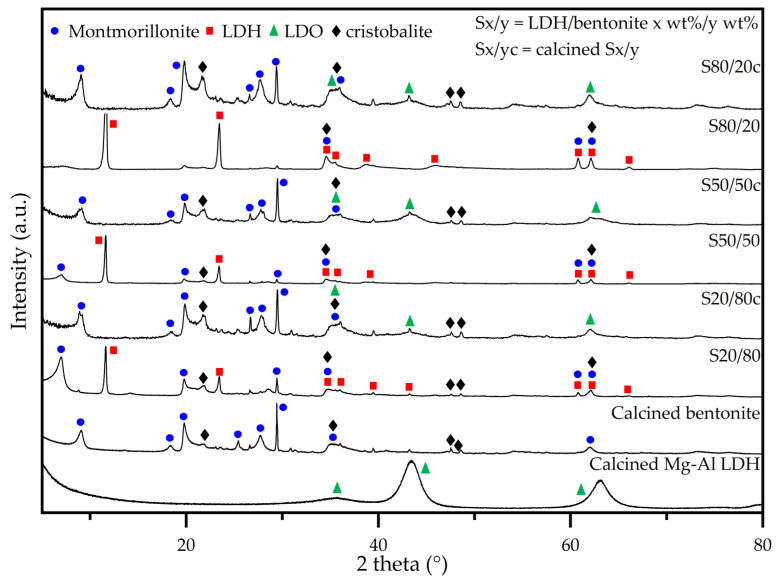
X-ray diffractograms of the (non)-calcined LDH/bentonite composites: assignment of the characteristic planes.

The XRD patterns of the as-synthesized adsorbents are represented in Figure 2 and for clarity reasons, the signals that also correspond to quartz (i.e., an additional component of bentonite) are not indicated. All non-calcined adsorbents display signals characteristic of both LDH and bentonite phases [46,47,48]. Both clay types exhibit XRD basal at low 2 theta angle and non-basal planes at higher 2 theta angles, respectively. First, the XRD basal planes are corresponding to their layered structure, the layers stacking one on top of each other creating an interlayer space where the water molecules and the ions are located, namely anions in case of LDHs and cations in case of bentonite. Secondly, the XRD non-basal planes correspond to the cation–cation distance within the brucite-like sheets of LDHs or bentonite, as well as other crystalline phases such as silica in the latter case, respectively. Upon calcination, the basal planes corresponding to LDH phase disappear as the structure is transforming into a mixed oxide (LDO) with concomitant loss of interlayer anions and water molecules. On the other hand, the basal planes corresponding to the bentonite phase tend to shift to higher 2 theta angle due to shrinkage of interlayer space as the water molecules are lost. Furthermore, it is also observed that with the increase in the LDH content in the composites, the bentonite reflections, especially the basal planes, diminish in intensity, for example barely being observed in the XRD pattern of the S80/20 non-calcined composite. This has to be regarded also taking into account that in the same series, the basal plane of LDH phase increases in intensity, consequently hindering the observance of the ones of bentonite. After calcination, the transformation of LDH phase on LDO structure leads to complete disappearance of the basal plane while in the case of bentonite these are shifted to a higher 2 theta angle due to shrinkage of the structure, as explained above. These also become visible in the XRD patterns of composites with increased bentonite content, as their presence is not hindered anymore by the presence of the LDH characteristic reflections. As can be observed, there is no (significant) change in the bentonite d_(001)_-non-basal spacing in the calcined samples when increasing the bentonite fraction, which suggests that the LDH is not incorporated in between the bentonite layers [49]. Additionally, there is no observable change in the lattice parameters (Table 3) (i.e., a and c), thus indicating the absence of a structural change in both the bentonite and LDH phases in the non-calcined granulates. As previously stated by Rodriguez et al. and Kim et al., an increase in the full width at half maximum (FWHM) indicates the occurrence of stacking disorder [35,50]. Thus, based on the decreasing FWHM with the increasing bentonite fraction, it can be concluded that the addition of the LDH negatively affects the stacking of the bentonite. An increase in the FWHM of the bentonite occurs upon calcination, which may be associated with an increase in the stacking disorder caused by dehydroxylation and dehydration of the interlayer of bentonite. Notably, a higher fraction of LDO (i.e., the calcined LDH) results in a better stacking order, which thus limits the collapse of the bentonite structure. Moreover, the signals of the bentonite (i.e., montmorillonite and cristobalite) remain present in the diffractograms after calcination [51,52,53]. Furthermore, upon calcination, the characteristic LDH peaks disappear and are replaced by signals characteristic of the layered double oxide (LDO), i.e., the calcined LDH, and this LDH to LDO conversion also has an impact on the d-spacing and the lattice parameters [54,55]. 

**Figure 3:**
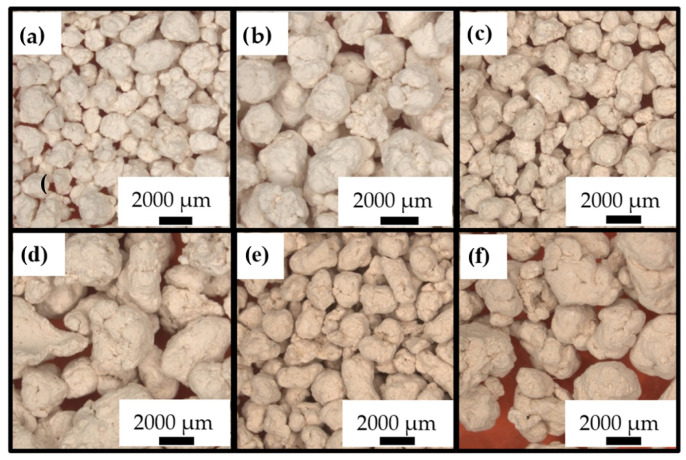
(**a**) Optical microscopy images of S80/20c—1–2 mm; (**b**) optical microscopy images of S80/20c—2–4 mm; (**c**) optical microscopy images of S50/50c—1–2 mm; (**d**) optical microscopy images of S50/50c—2–4 mm; (**e**) optical microscopy images of S20/80c—1–2 mm; (**f**) optical microscopy images of S20/80c—2–4 mm.

Moreover, a particle size study of the shaped granulates is necessary, since this influences the packing of the adsorbents and consequently also the flow rate, the reactant–adsorbent contact, the temperature and the pressure drop. Therefore, optical microscopy (OM) images and average particle sizes are included in Figure 3 and Table 4, indicating that the granulates have an average particle size within the specified range.

### 2.2. Sorption Study and Process Optimization

#### 2.2.1. Screening Adsorption Performance and Sorbent Selection

**Figure 4:**
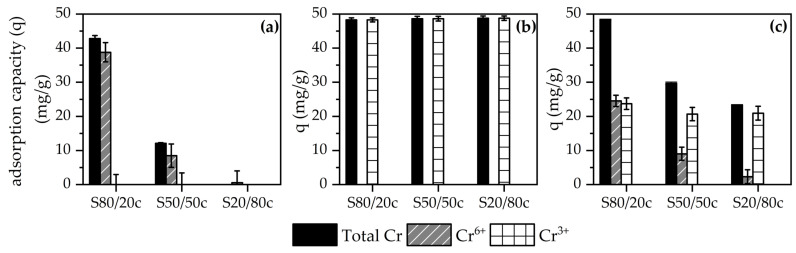
(**a**) Adsorbed amount of total Cr, Cr^6+^ and Cr^3+^ in a 200 ppm Cr^6+^ solution; (**b**) adsorbed amount of total Cr, Cr^6+^ and Cr^3+^ in a 200 ppm Cr^3+^ solution; (**c**) adsorbed amount of total Cr, Cr^6+^ and Cr^3+^ in a solution of 100 ppm Cr^6+^ and 100 ppm Cr^3+^.

As described in the previous sections, the granulated sorbents were designed having different compositions in terms of LDH/bentonite ratios aiming at the simultaneous sorption of both Cr^3+^ and Cr^6+^ species. To assess and select the ideal sorbent composition, preliminary screening tests were performed by contacting the crushed adsorbents with Cr^3+^, Cr^6+^ or a combination of Cr^3+^/Cr^6+^ solutions. Subsequently, the adsorption capacity can be calculated based on the Cr^3+^ or Cr^6+^ concentration before and after contact with an adsorbent, providing information about the performance of the sorbents. The results of the first screening tests are represented in Figure 4a–c, with pH values being included in Appendix B (Table A2). As evidenced by Figure 4a,c, the S80/20c adsorbent performs best for the adsorption of Cr^6+^ and an inversely proportional relationship can be observed between the amount of bentonite and the Cr^6+^ adsorption capacity. The LDH phase mainly targets the Cr^6+^ species due to its anionic nature, thus causing the higher Cr^6+^ adsorption of S80/20c. Furthermore, all adsorbents display a similar Cr^3+^ adsorption capacity (Figure 4b,c), indicating that the maximum Cr^3+^ adsorption capacity is possibly already reached in the S80/20c sample. Cr^3+^ could have precipitated at a pH of 6–9 in the form of Cr(OH)_3_, which dissolves again at a pH > 9 and then competes with the Cr^6+^-containing species [56]. Alternatively, if the calcined LDH/bentonite adsorbents have a sufficiently high standard electrode potential, i.e., E° > 1.35, then Cr^3+^ can be oxidized into Cr^6+^ [57]. Additionally, it can be observed that a mixture of both Cr^6+^ and Cr^3+^ results in a decrease in the adsorption of both compounds, which might be caused by repulsion between the adsorbed ions and the oppositely charged dissolved ions, preventing further diffusion into the pores. Alternatively, this decrease can be the result of the concentration change of both the Cr^6+^ and Cr^3+^ ions, which can affect the adsorption capacity of the adsorbents. Moreover, the pH might alter the charges and speciation of the composite material and chromium ions, respectively, which can also impact the adsorption capacity. Finally, it can be concluded that the S80/20c adsorbent displays the best overall performance, based on the results obtained during batch-mode experiments. Namely, the sample is characterized by a higher Cr^6+^ adsorption capacity due to the larger LDH fraction, and its overall performance excels in the experiments with mixtures of Cr^6+^ and Cr^3+^ species. Additionally, the better overall performance can be related to the higher specific surface area, the larger total pore volume, and the better stacking order of the composite material, as previously observed during the N_2_ sorption and XRD measurements.

**Figure A2:**
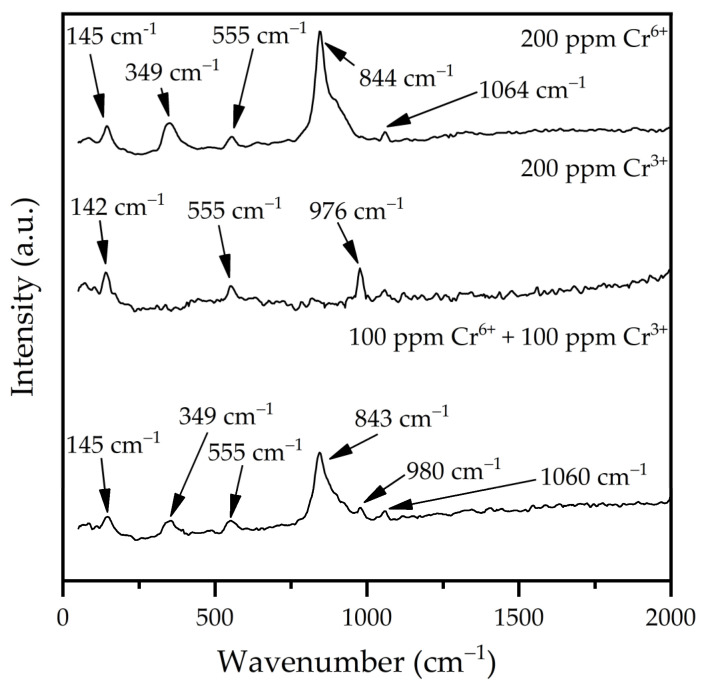
Structural characterization using Raman spectroscopy performed on the S80/20c sample after the adsorption tests.

Structural characterization performed on the adsorbent S80/20c after the sorption tests demonstrate that the Cr species are adsorbed by the composite adsorbent. When using Cr^6+^-containing solutions, the Raman spectrum (see Appendix B, Figure A2) shows the appearance of an intense peak around 843–844 cm^−1^, suggesting the successful adsorption of Cr^6+^-containing compounds onto the S80/20c sample. Indeed, the signal originates from the symmetric ν_1_(A_1_) vibration of CrO_4_^2−^, which is the dominant speciation of Cr at the solution pH [58,59,60]. This conclusion is further supported by the increasing intensity of the signal with the increase in Cr^6+^ concentration. The signal around 976–980 cm^−1^ might be caused by the ν_1_ symmetric stretching modes of SO_4_^2−^ [61,62,63,64,65]. Namely, this signal is only in the adsorbents that were tested in Cr^3+^-containing solutions. Moreover, in the Cr^6+^- and Cr^3+^-containing solution, the signal is less intense, suggesting that the adsorbent is more selective towards Cr^6+^ species compared to the sulphate anions. Due to the cationic nature of the Cr^3+^ species, its characteristic signals cannot be observed in the Raman spectra. However, due to the observed decrease in Cr^3+^ concentration during the batch-mode adsorption tests, it can be concluded that Cr^3+^ compounds were removed from the solutions.

**Figure 5:**
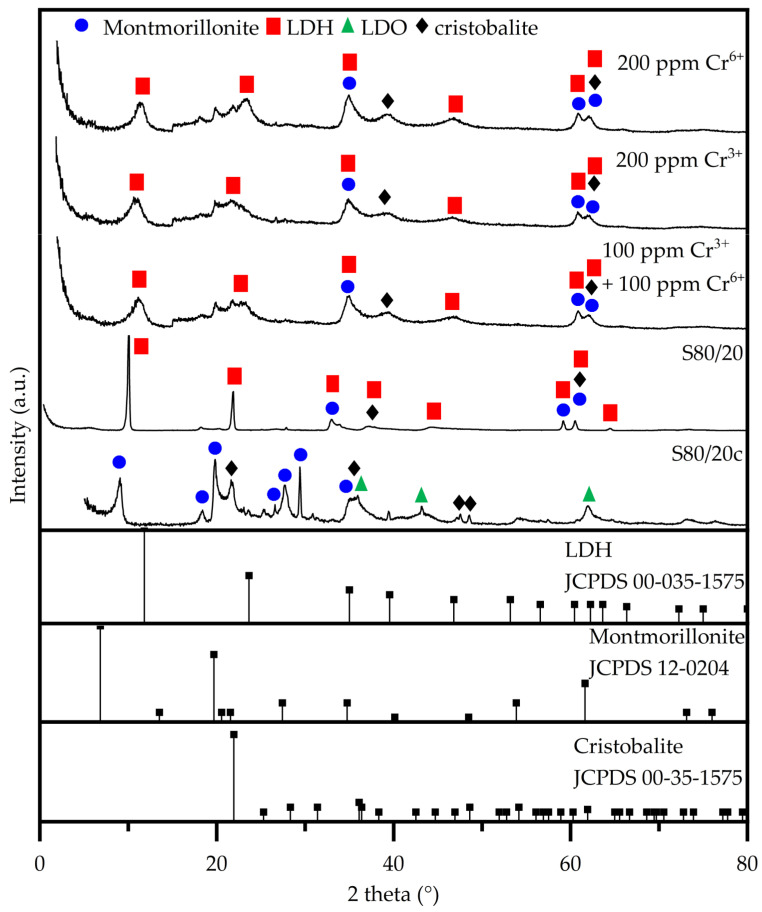
Structural characterization using XRD performed on the S80/20c sample after the adsorption tests.

The XRD characterization (Figure 5) demonstrates that after sorption, the LDH structure is reconstructed based on the unique memory effect property of the LDHs. This process involves a dissolution–recrystallization mechanism that leads to the appearance of the lower intensity and broader reflections assigned to the (003) plane of the LDH phase cantered at ~11° in the XRD patterns. Furthermore, the adsorption of the chromium species in between the interlayer regions results in a shift in the (003) plane, accompanied by a slight increase in d_(003)_ spacing (see Appendix B, Table A2). 

Based on these observations, the sorbent S80/20c was selected for further study and optimization of the chromium adsorption and desorption process. Additionally, as all the sorbent compositions adsorb similar amounts of Cr^3+^ species (see Figure 4), the further optimization study was focused on Cr^6+^ sorption.

#### 2.2.2. Kinetic Study

**Figure 6:**
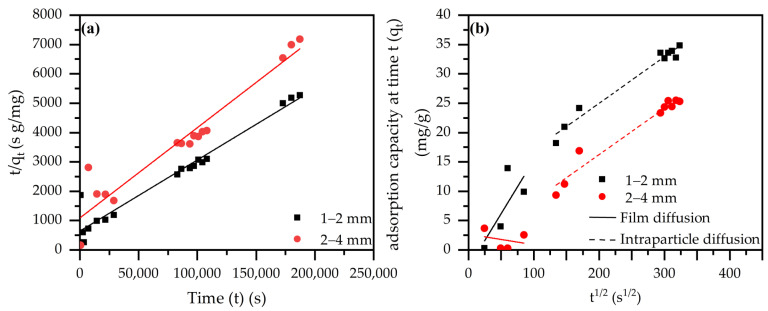
(**a**) Modeling of adsorption kinetics: pseudo-second order model S80/20c (C_i_ = 200 ppm Cr^6+^, S/L: 2.5 g/L); (**b**) modeling of the adsorption kinetics: intraparticle diffusion model (C_i_ = 200 ppm Cr^6+^, S/L ratio: 2.5 g/L).

Kinetic studies were performed for both size fractions of the S80/20c sample, the results of which are presented in Figure 6a,b and Table 5. The fastest adsorption kinetics are observable for the smallest size fraction (i.e., 1–2 mm), which is caused by the higher amount of available active sites compared to the larger size fraction (i.e., 2–4 mm). However, it is expected that equilibrium will be reached faster with the smallest size fraction, since smaller sizes are advantageous for diffusion. This hypothesis is supported by the observation that both the pseudo-first and pseudo-second order rate constants, k_1_ and k_2_, respectively, are larger for the smallest size fraction, suggesting its faster kinetic uptake. Furthermore, the pseudo-first and pseudo-second order models were used to generate estimates with reference to the equilibrium adsorption capacity. As it can be derived from the R^2^ values, the pseudo-second order model fits better for both size fractions. Moreover, the nonlinearity of the plots in Figure 6b suggests that the sorption process is controlled by multiple mechanisms. Namely, the first step is characteristic for film diffusion, while the second stage is associated with intraparticle diffusion. Additionally, the higher value of I (i.e., the external resistance for mass transfer from bulk across boundary layer to adsorbent surface) for the 1–2 mm size fraction indicates a higher amount of surface adsorption, but this does not influence the observed rate of diffusion (k_ID_). In addition, the Elovich model suggests the presence of a chemical interaction between the Cr^6+^ species and the composite surface. 

#### 2.2.3. Desorption Study and Process Optimization

Desorption experiments are essential for correctly evaluating the applicability of the adsorbents in a real-life setting and to enable their sustainable use in multiple adsorption/desorption cycles. During, e.g., chromium tanning, only 60–70% of the total chromium salts react with animal hides and skin, resulting in a loss of approximately 30–40% [66]. In this light, the chromium pollution also has an economic impact, which can be counteracted using chromium recovery through desorption. However, despite its importance, chromate desorption studies on LDH materials are scarcely found in the state-of-the-art. Lazaridis et al. previously investigated the impact of the eluant concentration and volume while also providing a kinetic study [43]. However, the impact of the desorption solution pH was not evaluated, and the kinetic study did not consider the Elovich equation or the IPD model. Furthermore, Bayuo et al. investigated different desorption solutions while disregarding the kinetics and the effect of the pH and concentration [67]. Otero et al., on the other hand, did investigate the influence of pH on desorption, but the influence of concentration, the kinetics and adsorption–desorption cycles were not investigated [68]. Consequently, it is important to analyze the effect of all possible parameters that might have an impact on desorption.

**Figure 7:**
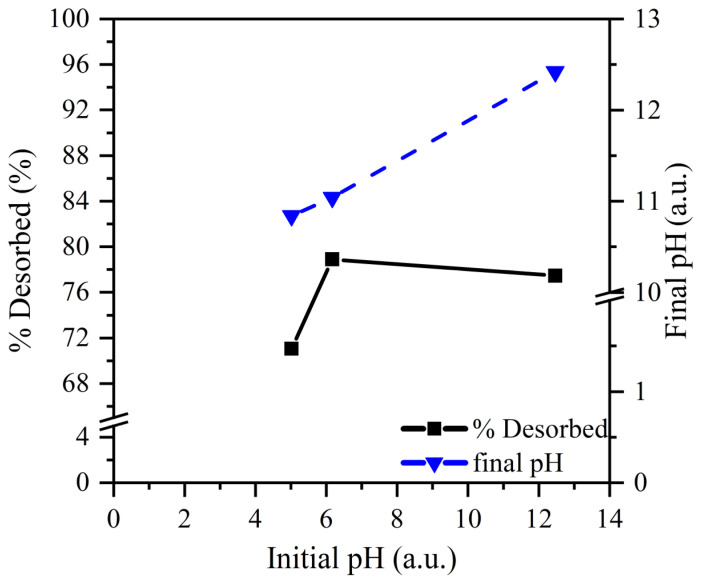
Influence of the initial pH on the Cr^6+^ desorption from S80/20c—1–2 mm (S/L ratio: 2.5 g/L (adsorption step); S/L ratio: 5 g/L (desorption step); C_NaCl_ = 1 M, contact time of 24 h).

**Figure 8:**
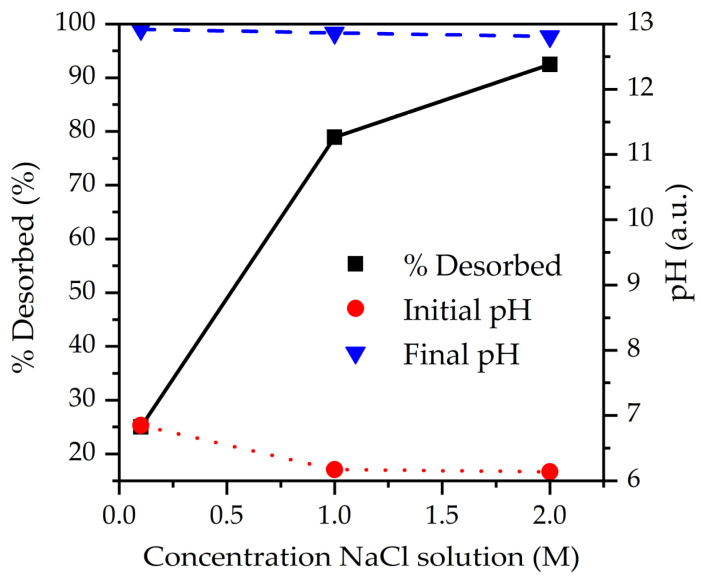
Influence of the initial Cl^−^ concentration on the Cr^6+^ desorption from S80/20c—1–2 mm; S/L ratio: 2.5 g/L (adsorption step) and S/L ratio: 5 g/L (desorption step), contact time of 24 h, pH = neutral.

To optimize desorption conditions, the influence of pH was evaluated while maintaining the NaCl concentration constant. As can be observed from Figure 7, pH does not greatly affect desorption efficiency at 1 M NaCl. Optimum desorption efficiency is observed at pH 6, while an increased pH above 6 does not further improve the desorption efficiency. Moreover, when considering Figure 8, a strong correlation can be observed between the initial NaCl concentration and the desorption percentage, resulting in the conclusion that Cl^−^ concentration is the main trigger of desorption. Additionally, Cl^−^ ions can compete with CrO_4_^2−^ species due to their negative charge.

**Figure 9:**
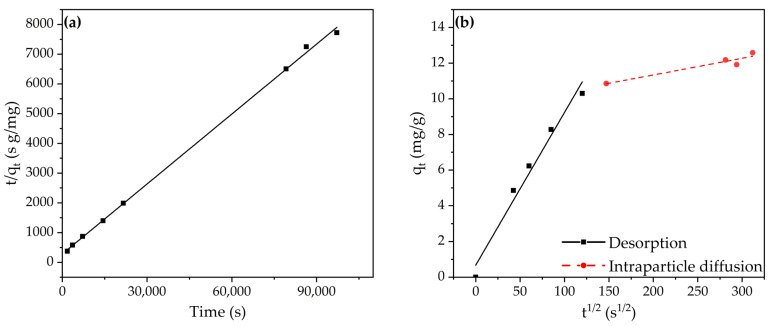
(**a**) Modeling of desorption: pseudo-second order model S80/20c—1–2 mm (2 M NaCl; pH 6.14; S/L ratio: 5 g/L); (**b**) modeling of desorption: intraparticle diffusion model (2 M NaCl; pH 6.14; S/L ratio: 5 g/L).

Similarly, as for the adsorption tests, the kinetics were evaluated for the desorption study. The equilibrium desorption capacity was estimated using the pseudo-first and pseudo-second order models, which can be found in Table 6. Based on the obtained R^2^, it can be concluded that the pseudo-second order model is a better fit for the desorption kinetics. Notably, the adsorption and desorption kinetics of the 1–2 mm size fractions are both represented by this model. Furthermore, Figure 9b also indicates the two-stage mechanism of the desorption process, with the first stage being representative of the desorption of the Cr^6+^ species from the adsorbent surface. Subsequently, the desorption is followed by the diffusion of the Cr^6+^ compounds away from the adsorption sites and consequently through the pores of the composite material in the direction of the bulk solution. As can be observed from the desorption rate constants, the intraparticle diffusion—i.e., the second stage—can be designated as the rate-limiting step.

#### 2.2.4. Multicycle Process

In order for an adsorbent to be usable in real-life applications, it should show good performance over multiple adsorption–desorption cycles. Consequently, this has already been investigated in the state-of-the-art by, e.g., Wang et al., Xu et al. and Bayuo et al. [37,42,67]. However, in multiple cases, the adsorption and desorption percentage decreases with an increasing number of adsorption–desorption cycles, suggesting room for improvement [37].

**Figure 10:**
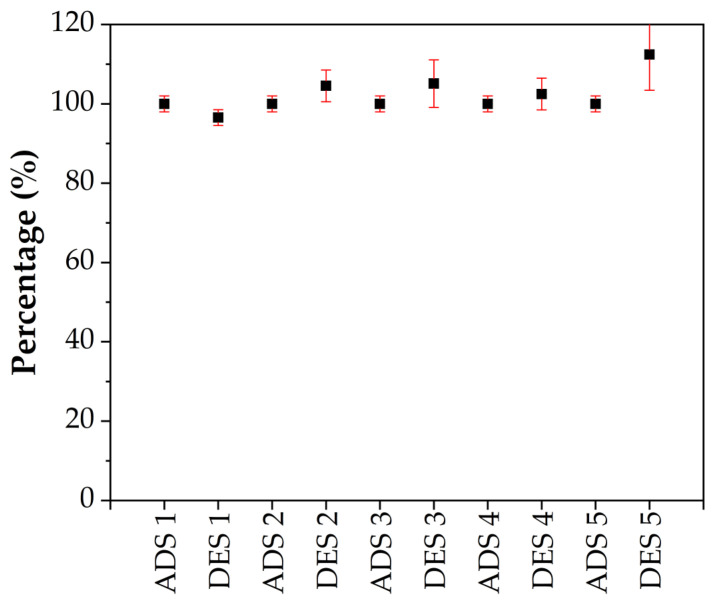
Adsorption (ADS) and desorption (DES) cycles using S80/20c—1–2 mm (S/L ratio: 2.5 g/L (adsorption step); S/L ratio: 5 g/L (desorption step); C_NaCl_ = 2 M; contact time 24 h).

Consequently, to further assess the applicability of S80/20c—1–2 mm in industry, the adsorbent was evaluated for its reuse potential. In this regard, five adsorption–desorption cycles were performed, resulting in the data presented in Figure 10. The adsorption was performed in an analogous way as before, with 0.25 g of the adsorbent being contacted with 100 mL of a 100 ppm Cr^6+^ solution. The desorption cycles, however, were performed using the optimized desorption conditions, i.e., using a 2 M NaCl desorption solution with a pH of 6.14. An adsorption and desorption percentage of approximately 100% was achieved, clearly indicating the outstanding performance of the adsorbent. No decline was observed during the successive adsorption or desorption cycles, resulting in the conclusion that the adsorbent is characterized by excellent stability. Additionally, it should be mentioned that the adsorbent is an LDH/bentonite composite after the first cycle since the recovered adsorbent is not calcined in between two adsorption–desorption cycles. As can be concluded from Figure 10, this Cl^−^-containing LDH/bentonite performs equally well or even better than the calcined adsorbents.

## 3. Materials and Methods

### 3.1. Materials

Pural MG63 HT was provided by Sasol Germany GmbH, while bentonite was purchased at VWR Chemicals. Pural MG63 HT is a MgAl-LDH type material, with its chemical formula being Mg_0.667_Al_0.333_(OH)_2_(CO_3_)_0.167_(H_2_O)_0.5_. The Mg/Al ratio and the apparent surface area are equal to 2 and 20 m^2^/g, respectively. Moreover, the particle sizes vary up to 38 µm (d_10_ of 2.7 μm, d_50_ of 10.2 μm and d_90_ of 37.5 μm). The commercial bentonite Na form CAS 1302-78-9, on the other hand, consists of 95.5 wt% bentonite, 2.5 wt% quartz and 2% sodium carbonate. Cr_2_(SO_4_)_3_.xH_2_O CAS 15244-38-9 and Na_2_CrO_4_ 98%, CAS 7775-11-3; NaCl ACS reagent, ≥99.0% CAS 7647-14-5, 0.01M HCl ACS reagent; 37% CAS 7647-01-0 and 0.1M NaOH ACS reagent, ≥97.0% CAS 1310-73-2 were purchased at Sigma-Aldrich, St. Louis, MO, USA. Finally, colloidal silica (Ludox HS-40, 40 wt.% suspension in H_2_O, Na stabilized) was obtained from Grace GmBH, Worms, Germany.

### 3.2. Preparation of the Mg-Al LDH/Bentonite Adsorbents

The aim of the granulation step was to form composite granulates of MgAl LDH and bentonite, preferably with a diameter within the range of 1–4 mm. Granulation experiments were carried out using an intensive mixer (EL5 Profi Plus, Eirich, Hardheim, Germany). The composition of the three different formulations used is shown in Table 2. Distilled water was used as a granulation liquid (no other binder was used) during the granulation process. Water was added during stirring to promote particle agglomeration and packing. Silica Ludox (2 wt%) was added to samples with a larger content of bentonite to improve the mechanical strength of the obtained granulates. Water was added in several steps to avoid the formation of big wet lumps. The rotation speed of the bowl was set to 40 rpm and was kept constant during the granulation experiment. The rotation speed of the impeller was set to 3500 rpm during the addition of water (ca. 30 s) and to 1500 rpm during the sphere growing process (ca. 5 min). These parameters were determined based on preliminary granulation tests. After granulation, the produced spheres were dried at 40 °C for 24 h followed by a calcination treatment to achieve their final mechanical strength. Calcination treatment was performed for 4 h at 550 °C, starting from 25 °C with a heating rate of 1 °C/min. The cooling was subsequently performed using a cooling rate of 1 °C/min. After calcination, the produced granulates were sieved using laboratory sieves to four size fractions: <1 mm, 1–2 mm, 2–4 mm and >4 mm. For further investigation, only the size fractions of 1–2 mm and 2–4 mm were used. The obtained samples were labelled Sx/y or Sx/yc with x/y representing the Mg-Al LDH/bentonite ratio, and c representing calcination.

### 3.3. Characterization

A Horiba Xplora Plus Microscope Raman spectrometer, purchased from Acal Technology Netherlands, was used for the evaluation of the Raman-active vibrational modes of the bonds present in the sample. A laser with a wavelength of 532 nm was used for the measurements and the granulates were manually crushed before performing the Raman spectroscopy measurements. Raman results can be found in Appendix A and Appendix B.

Hg intrusion measurements were performed using a PASCAL 140 porosimeter and a PASCAL 240 porosimeter, Thermo Fisher Scientific, Rodano, Italy. The PASCAL 140 porosimeter is used for the evaluation of ultramacro- and macroporosity, while the PASCAL 240 porosimeter provides pore size and volume information in a radius range of 3.7 nm to 7500 nm at high pressure.

N_2_ physisorption was performed by degassing the samples for 16 h at a temperature of 200 °C in an AS-6 degasser. This was followed by measurements using a Quantachrome Quadrasorb SI, and the Brunauer–Emmet–Teller (BET) equation was used to calculate the specific surface area. Pore size distribution was subsequently estimated from the desorption branch using the Barret–Joyner–Halenda method, while the total pore volume was determined from the adsorption branch at a P/P_0_ of 0.98.

All adsorbents were analyzed in a 2θ range of 5–80° using the PANalytical X’Pert PRO MPD, Malvern Panalytical, Amelo, Nederlands, which used Cu K_α_ rays (λ = 0.15406 nm) and a scan speed of 0.04°/4 s. Additionally, the used Sx/y samples (i.e., the adsorbents retrieved after the batch-mode adsorption experiments) were measured between 0.4° and 14.98° using the bracket setup, and the PANalytical ICSD database was used to process the data.

Optical microscopy was performed using the optical microscope ZEISS Stereo Discovery V12, in combination with a PlanAPO S 1.0× (FWD = 60 mm) objective lens, a ZEISS Axiocam 506 colour camera and a ZEISS KL 2500 LCD light source. All Optical equipment is acquired from Zeiss, Oberkrochen, Germany.

### 3.4. Sorption Study and Process Optimization

Chromium batch-mode adsorption experiments were performed in duplicate by adding the manually crushed Sx/yc to Cr^3+^ and/or Cr^6+−^containing solutions, which were prepared using Cr_2_(SO_4_)_3_.xH_2_O and Na_2_CrO_4_. The stock solutions were added to the weighed and crushed adsorbents in a 2.5 g/L S/L ratio, after which the entirety was stirred for up to 50 h at 300 rpm. It is important to note that these tests were performed on the crushed adsorbents for composition screening purposes only, i.e., selection of best-performing Mg-Al LDH/bentonite ratio. Consequently, during subsequent experiments, tests were performed on the granulates. The liquid, which was filtered using a 0.45 µm filter, was evaluated for total chromium adsorption using ICP-OES analysis (Agilent type 5100 VDV, radial torch). Hexavalent chromium adsorption was additionally evaluated using the Chromium(VI) High range method of the HI883300 multiparameter photometer, Hanna Instruments, Temse, Belgium. The adsorbed chromium concentrations were calculated using Formulas (1)–(3), with Cr_initial_ (ppm) and Cr_final_ (ppm) representing the chromium concentration in the initial and final solution, respectively. Furthermore, m_adsorbent_/V_liquid_ (g/L) equals the S/L ratio and q (mg/g) represents the adsorption capacity of the adsorbent.

(1) $$q\left({\mathrm{Cr}}^{3+}{+\mathrm{Cr}}^{6+}\right)=\frac{\left({\mathrm{Cr}}_{\mathrm{initial}}^{\mathrm{total}}-{\mathrm{Cr}}_{\mathrm{final}}^{\mathrm{total}}\right){\;\times \;V}_{\mathrm{liquid}}}{m_{\mathrm{adsorbent}}}$$

(2) $$q\left({\mathrm{Cr}}^{6+}\right)=\frac{\mathrm{dilution}\;\mathrm{factor}\;\times \;\left({\mathrm{Cr}}_{\mathrm{initial}}^{6+}-{\mathrm{Cr}}_{\mathrm{final}}^{6+}\right){\;\times \;V}_{\mathrm{liquid}}}{m_{\mathrm{adsorbent}}}$$

(3) $$q\left({\mathrm{Cr}}^{3+}\right)=c\left({\mathrm{Cr}}^{3+}{+\mathrm{Cr}}^{6+}\right)-\;c\left({\mathrm{Cr}}^{6+}\right)$$

The collected solids were subsequently washed twice with deionized water, after which the powder was dried at 40 °C. Raman spectroscopy and XRD were subsequently performed on the best-performing adsorbent (i.e., S80/20c after adsorption).

The kinetics of the Cr^6+^ adsorption was additionally studied by monitoring the adsorbed Cr^6+^ amount in function of the contact time. The study of the adsorption kinetics aids in the understanding of the adsorption mechanism and is additionally used for the design of columns at the laboratory, pilot and plant scales [69,70]. Here, the experiments were performed using a tank and a magnetic stirrer, i.e., a slurry batch reactor [69]. A 1000 mL solution containing 200 ppm Cr^6+^ was initially prepared using Na_2_CrO_4_, after which 2.5 g of a size fraction of S80/20c was added. The mixture was shaken on a shaking table at 100 rpm for 52 h and 2 mL of the solution was sampled at designated time points. Finally, the filtered liquid (used filter: 0.45 µm) was analyzed using the HI883300 multiparameter photometer. Pseudo-first order, pseudo-second order, Elovich and intraparticle diffusion (IPD) models were additionally used to model the collected data. The collected granulates were additionally washed twice with deionized water and dried at 40 °C.

Based on the fastest adsorption kinetics, the 1–2 mm size fraction of the S80/20c sample was selected for desorption tests using concentrated NaCl solutions. Firstly, the granulates were loaded with Cr^6+^ by placing 0.25 g in a setup with a filter mesh. One hundred milliliters of a 100 ppm Cr^6+^ solution was added to the granulates and the entirety was stirred for 24 h at 500 rpm. Furthermore, the solids were washed twice using 40 mL of deionized water while stirring for 10 min, after which they were added to 50 mL of the different desorption solutions. The mixtures were stirred for 24 h. Different desorption solutions were tested for the investigation of the effect of NaCl concentration (0.1 M NaCl, 1 M NaCl or 2 M NaCl at pH 7) and effect of pH (pH 5, 7 and 12.5). All solutions were filtered using a 0.45 µm filter and analyzed using an HI883300 multiparameter photometer.

### 3.5. Desorption Optimization and Process Optimization

The desorption kinetics were additionally analyzed by adding 0.5 g of the 1–2 mm size fraction of S80/20c and 100 mL of a 100 ppm Cr^6+^ solution to a setup using a filter mesh. The mixture was stirred for 24 h at 500 rpm. Thereafter, the loaded solids were washed twice using 40 mL of deionized water while stirring for 5 min. The wet solids were subsequently added to 100 mL of the 2 M NaCl (unaltered pH) desorption solution and the entirety was stirred at 500 rpm for 24h. Filtered samples (used filter: 0.45 µm) were collected at designated times and analyzed using an HI883300 multiparameter photometer.

### 3.6. Multicycle Process

Five adsorption and desorption cycles were performed to evaluate the recyclability of the S80/20c adsorbent. A quantity of 0.25 g of the 1–2 mm size fraction and 100 mL of the 100 ppm Cr^6+^ solution were combined in a setup containing a mesh filter. The mixture was stirred for 24 h at 500 rpm to allow the Cr^6+^ adsorption onto the granulates. The obtained granulates were washed twice using 40 mL of deionized water while stirring for 5 min. Next, Cr^6+^ desorption was performed by adding 50 mL of the 2 M NaCl solution and stirring again for 24 h. Finally, the granulates were washed twice using 40 mL of deionized water while stirring for 5 min. This adsorption–desorption cycle protocol was performed for a total of five cycles. All the obtained solutions, e.g., after adsorption and desorption, as well as all the washing solutions, were analyzed using an HI883300 multiparameter photometer.

## 4. Conclusions

Mg-Al LDH/bentonite composites with different LDH/bentonite ratios were prepared in granulated form and their material properties were characterized. The granulates were calcined to achieve their mechanical stability, after which composites of Mg-Al LDO/bentonite were obtained. The composites were designed for the adsorption of both Cr^3+^ and Cr^6+^ species, with high capacity and regeneration abilities to enable their multicycle use in real-life applications. The composite with the LDH/bentonite ratio of 80/20, e.g., composite denoted S80/20c, ratio displayed the most promising results for the chromium abatement (especially for Cr^6+^) and this was further used for process optimization and kinetic study in both adsorption and desorption steps. The granulates showed slower kinetics for Cr^6+^ adsorption compared with the powders, reaching the equilibrium capacity within 23 h, respectively, but still with ~70% of Cr^6+^ adsorbed in the first period of adsorption process. Both the adsorption and the desorption kinetic data modelling indicated that the process follows the pseudo-second order model, indicating a chemical interaction sorption mechanism. Desorption using NaCl at different pH and concentrations was investigated and the results showed that a 2 M NaCl solution with a natural pH of 6.14 was the most optimal for multicycle use. This resulted in the excellent performance and stability of the 1–2 mm size fraction of the S80/20c adsorbent during five consecutive adsorption–desorption cycles without any loss in its adsorption and desorption capacity. Consequently, the developed calcined LDH/bentonite composites clearly show high potential for the abatement and recovery of chromium in wastewater purification or resource recovery applications. Moreover, their granulated shape enables their integration in column or other designs of installations and they are easy to handle and separate at the end of the process, all these aspects being of great importance in practical applications.

## Figures and Tables

**Table 1:** Examples of state-of-the art layered double hydroxides (LDHs), bentonite and LDH/bentonite adsorbents for the removal of chromium species.

Adsorbent	Max. Cr^3+^ Adsorption (Units as Reported)	Max. Cr^6+^ Adsorption (Units as Reported)	Solid/Liquid Ratio S/L (g/L)	Reference
Bentonite	100% (pH 7)	40% (pH < 1.5)	1/20	[24]
Zeo-bentonite	-	±81%	10	[25]
Chitosan/bentonite	-	16.38 mg/g	10	[26]
Montmorillonite	-	785.14 mg/g	40	[27]
Algerian bentonite	-	12.61 mg/g	25	[28]
Bentonite	151.51 mg/g	161.29 mg/g	1	[29]
Bentonite@MnFe_2_O_4_	175.44 mg/g	178.57 mg/g	1	[29]
Bentonite	-	59.523 mg/g	1	[30]
Bentonite/bio-coal	-	64.102 mg/g	1	[30]
1CPC (modified bentonite)	-	24.83 mg/g	1	[31]
Mg-Al-Cl Layered double hydroxide (LDH)	-	45.20 mg/g	2	[32]
in-situ LDH	-	339 mg/g	-	[33]
Mg-Al-CO_3_ LDH	-	246 mg/g	2	[33]
Mg-Al-Fe LDO	-	725.61 mg/g	4	[34]
LDH-bentonite composite 50/50	-	6.705 mg/g	1	[35]
Bentonite-Co-Al LDH	-	101.06 mg/g	0.125	[36]
Zn/Al-CO_3_ LDH	-	68.07 mg/g	0.2	[37]
MgAl LDH	-	30.28 mg/g	0.2	[37]
Calcined graphene- Mg/Al LDH	-	172.55 mg/g	0.1	[38]
Oriented Mg/Al–NO_3_-LDH	-	8.2 mg/g	0.85	[22]
Mg/Al-LDH/ESM	-	27.9 mg/g	5	[23]
Li/Al-LDH	-	177 mg/g	2	[39]
Calcined Mg/Al–CO_3_-LDH	-	122 mg/g	0.2	[40]
CaAl-Cl LDH after synthesis	-	77.99 mg/g	4	[41]
Calcined MgAl-CO_3_-HT	-	34.3–44.7 mg/g	(column test)	[42]
Mg-Al-CO_3_ hydrotalcite	-	17 mg/g	1	[43]
Acid-activated kaolinite	-	13.9 mg/g	2	[44]
Exfoliated LCT-LDH	-	125.97 mg/g	2	[45]
CS/Clay/Fe_3_O_4_	-	117.64 mg/g	1	[17]

**Table 2:** Characteristics of the calcined starting materials (i.e., bentonite and Mg-Al LDH) and adsorbents.

Property	Calcined LDH/Bentonite 80 wt%/20 wt% (S80/20(c))	Calcined LDH/Bentonite 50 wt%/50 wt% (S50/50(c))	Calcined LDH/Bentonite 20 wt%/80 wt% (S20/80(c))	Calcined Bentonite	Calcined Mg-Al LDH
^1^ Mg-Al LDH, wt%	51.7	32.4	13.4	-	100
^1^ Bentonite, wt%	12.9	32.4	53.7	100	-
^1^ Colloidal silica, wt%	-	2.0	2.0	-	-
^1^ Water, wt%	35.4	33.2	30.9	-	-
^1^ Rotor speed, m/s	3	4	3	-	-
^1^ Vessel speed, m/s	0.5	0.5	0.5	-	-
^1^ Mixing time, s	420	340	315	-	-
Specific surface area (S_BET_) (10^−3^ m^2^/kg)	183 ± 18	104 ± 10	33 ± 3	38 ± 4	187 ± 19
Total pore volume (10^−3^ m^3^/g)	0.23 ± 0.02	0.18 ± 0.02	0.10 ± 0.01	0.08 ± 0.01	0.07 ± 0.01
^2^ Pore radius (d_p_) range (Å)	17–22 ± 2	15–23 ± 2	18–23 ± 2	17–23 ± 2	17–20 ± 2

^1^ Conditions used during the adsorbent preparation; ^2^ Pore radius range determined through the Barrett–Joyner–Halenda (BJH) method applied to the desorption branch of the isotherm at p/p_0_ = 0.98.

**Table 3:** Calculated d-spacings (in nm) and full width at half maximum (FWHM) of the adsorbents.

Sample	^1^ d_(001)_ (nm)	^2^ a (nm)	^1^ c (nm)	^3^ FWHM (°)	^4^ d_(003)_ (nm)	^5^ a (nm)	^4^ c (nm)	^6^ FWHM (°)
Calcined Mg-Al LDH	-	-	-	-	-	-	-	-
Calcined bentonite	0.97	0.90	0.97	1.37	-	-	-	-
LDH/bentonite 80 wt%/20 wt% (S80/20)	1.22	0.90	1.28	2.29	0.76	0.30	2.27	0.22
S80/20c	1.00	0.91	1.00	0.84	-	-	-	-
LDH/bentonite 50 wt%/50 wt% (S50/50)	1.28	0.90	1.28	1.87	0.75	0.30	2.26	0.21
S50/50c	0.96	0.90	0.96	1.92	-	-	-	-
LDH/bentonite 20 wt%/80 wt% (S20/80)	1.28	0.90	1.22	1.55	0.76	0.30	2.29	0.22
S20/80c	1.00	0.90	1.00	2.26	-	-	-	-

^1^ Obtained using Bragg’s law for the bentonite (001) plane; ^2^ obtained using the bentonite (110) plane; ^3^ full width at half maximum (FWHM) of the bentonite (001) plane; ^4^ obtained using Bragg’s law for the LDH (003) plane; ^5^ obtained using the LDH (110) plane; ^6^ FWHM of the LDH (003) plane.

**Table 4:** Average particle size obtained using optical microscopy for the size fractions of S80/20c, S50/50c and S20/80c.

Sample	Particle Diameter (mm)	
	1–2 mm	2–4 mm
S80/20c	2.17 ± 1.0	2.61 ± 1.9
S50/50c	1.55 ± 0.9	3.21 ± 2.0
S20/80c	1.75 ± 0.8	2.72 ± 2.0

**Table 5:** Kinetic parameters for the adsorption of Cr(VI) onto S80/20c.

Model	Parameter	S80/20c 1–2 mm	S80/20c 2–4 mm
Experiment	Initial concentration (C_i_) (ppm)	215.6 ± 10.7	202.4 ± 10.1
	Experimental equilibrium adsorption capacity (q_e_) (mg/g)	35.5 ± 2.8	26.5 ± 5.0
Pseudo-first order $q_{t}{=q}_{e}{(1-\exp(-k}_{1}t))$	Equilibrium adsorption capacity (q_e_) (mg/g)	35.5 ± 2.8	26.5 ± 5.0
	Reaction rate (k_1_) (1/s)	(2.3 ± 0.3) × 10^−5^	(2.5 ± 0.3) × 10^−5^
	R^2^	0.86	0.86
Pseudo-second order $\frac{t}{q_{t}}=\frac{1}{k_{2}q_{e}^{2}}+\frac{t}{q_{e}}$	Equilibrium adsorption capacity q_e_ (mg/g)	32.5 ± 2.6	41.3 ± 2.4
	Reaction rate (k_2_) (g/s mg)	(8.7 ± 2.0) × 10^−7^	(9.1 ± 1.9) × 10^−7^
	R^2^	0.93	0.95
Elovich equation $q_{t}=\frac{1}{\beta }\ln\left(1+\alpha \beta t\right)$	Initial adsorption rate (α) (mg min^−1^ g^−1^)	0.5 ± 0.3	0.3 ± 0.2
	Desorption constant (β) (mg/g)	0.15 ± 0.02	0.17 ± 0.03
	R^2^	0.96	0.89
Intraparticle diffusion model $q_{t}{=k}_{\mathrm{id}}t^{1/2}+I$	Reaction rate (k_1_) (mg s^−1/2^ g^−1^)	0.2 ± 0.1	−0.02 ± 0.0
	Rate of diffusion (K_ID_) (mg s^−1/2^ g^−1^)	0.1 ± 0.0	0.1 ± 0.0
	External resistance (I) (mg/g) (step 2)	9.2 ± 1.3	0.5 ± 1.7
	R^2^ (step 1)	0.60	0.10
	R^2^ (step 2)	0.97	0.96

**Table 6:** Kinetic parameters for the desorption of Cr^6+^ from the S80/20c adsorbent surface.

Model	Parameter	S80/20c 1–2 mm
Experiment	C_i_ (ppm)	100.6 ± 1.2
	q_e,des_ (exp) (mg/g)	12.6 ± 0.3
Pseudo-first order $q_{t}{=q}_{e}(1\;-\;\exp(-k_{1}t))$	q_e,des_ (mg/g)	12.58 ± 0.3
	k_1,des_ (1/s)	(3.16 ± 0.55) × 10^−5^
	R^2^	0.85
Pseudo-second order $\frac{t}{q_{t}}=\frac{1}{k_{2}q_{e}^{2}}+\frac{t}{q_{e}}$	q_e,des_ (mg/g)	12.75 ± 0.16
	k_2,des_ (g/s mg)	(2.17 ± 0.21) × 10^−5^
	R^2^	1.00
Elovich equation $q_{t}=\frac{1}{\beta }\ln\left({1+\alpha }_{\mathrm{des}}\beta_{\mathrm{des}}t\right)$	α_des_ (mg min^−1^ g^−1^)	2.08 ± 1.6
	β_des_ (mg/g)	1.94 ± 3.8
	R^2^	0.95
Intraparticle diffusion model $q_{t}{=k}_{\mathrm{ID},\mathrm{des}}t^{1/2}+I$	k_1,des_ (mg s^−1/2^ g^−1^)	0.09 ± 0.0
	K_ID,des_ (mg s^−1/2^ g^−1^)	0.01 ± 0.0
	I_des_ (mg/g) (step 2)	9.47 ± 0.53
	R^2^ (step 1)	0.98
	R^2^ (step 2)	0.92

**Figure A1:**
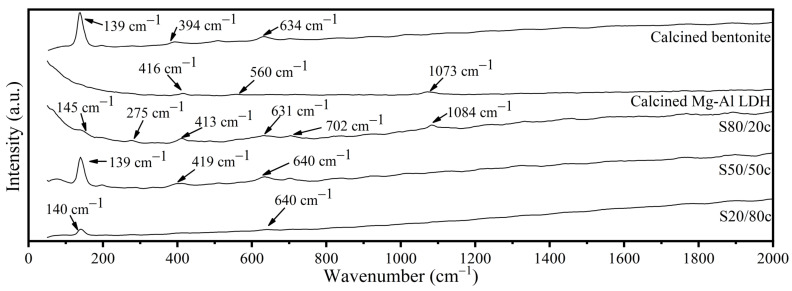
Structural characterization using Raman spectroscopy performed on the calcined starting materials (i.e., bentonite and MgAl LDH) and Sx/yc.

## Data Availability

The data presented in this study are available in “Structured LDH/bentonite composites for chromium removal and recovery from aqueous solutions”.

## Sample Availability

Samples of the compounds are not available from the authors upon request.

## Appendix A

The Raman spectra of the calcined starting materials and Sx/yc samples are represented in Figure A1. The calcined bentonite displays a signal around 139 cm^−1^, which is characteristic of the Si_2_O_5_ ring breathing mode of montmorillonite, the main component of bentonite [71,72]. Additionally, the signal around 394 cm^−1^ is probably caused by the ν_3_(a_1_) internal vibrational mode of the SiO_4_ tetrahedral units, while the signal at 634 cm^−1^ might be assigned to the Si-O-Si vibration mode [72,73]. Contrarily, the calcined Mg-Al LDH displays peaks at 416 cm^−1^, 560 cm^−1^ and 1073 cm^−1^. The signal at 416 cm^−1^ originates from Al-O-Al linkages, while the band at 560 cm^−1^ is caused by the metal-O bonds in the LDH structure [74,75,76,77,78,79]. Furthermore, the signal at 1073 cm^−1^ can be assigned to carbonate anions associated with either the hydroxyl surface or water, which is caused by the absence of an inert atmosphere during storage, thus allowing for the adsorption of CO_2_ and water [75,76,78,79,80,81]. S80/20c, on the other hand, is characterized by signals around 145 cm^−1^, 275 cm^−1^, 413 cm^−1^, 631 cm^−1^, 702 cm^−1^ and 1084 cm^−1^. According to Paikaray et al. and Frost et al., the peak at 145 cm^−1^ is caused by lattice vibrations or M-O vibrations, with M representing the divalent and trivalent metals [64,77]. However, the band can also be assigned to the ν_2_(E_2g_) mode of the AlO_6_ octahedron of montmorillonite while the signal at 275 cm^−1^ is in all probability the A_1_(ν_1_) mode of the O-H-O triangle present in montmorillonite [72]. The vibration at 413 cm^−1^ probably originates from either or both the ν_3_(a_1_) internal vibrational mode of the SiO_4_ tetrahedral units in the montmorillonite fraction and the M-O vibrations in the LDH material [72,76,77,78,79]. The Si-O-Si vibration mode is then responsible for the peaks at 631 cm^−1^ and 702 cm^−1^ and the latter signal can also be assigned to a metal–oxygen mode [73,82]. Finally, the band at 1084 cm^−1^ is caused by the ν_3_(f_2_) vibrational mode of the SiO_4_ units in montmorillonite [72]. Similarly, three bands can be observed for the S50/50c sample at 139 cm^−1^, 419 cm^−1^ and 640 cm^−1^. The signal with the lowest Raman shift can be assigned in an analogous way as for the calcined bentonite, while the signal at 640 cm^−1^ is characteristic of the Si-O-Si vibration mode in montmorillonite. Moreover, the band at 419 cm^−1^ originates from both the calcined LDH and bentonite, as explained before. Finally, two Raman peaks were detected for the S20/80c sample at 140 cm^−1^ and 640 cm^−1^ and both signals were already clarified in the discussion of the calcined starting materials.

**Table A1:** Hg intrusion porosity data of S80/20c 1–2 mm.

Sample	Total Cumulative Volume (mm^3^/g)	Average Pore Radius (µm)	Total Porosity (%)
S80/20c 1–2 mm	636	0.06	54
S80/20c 2–4 mm	608	0.06	59

## Appendix B

**Table A2:** pH variations during adsorption in a solution containing 200 ppm Cr^6+^, 200 ppm Cr^3+^ or 100 ppm Cr^6+^ and 100 ppm Cr^3+^ (S/L ratio: 2.5 g/L; contact time of 24 h).

Sample	Concentration	pH_initial_	pH_final_
S80/20c	200 ppm Cr^6+^	8.21	11.74
S80/20c	200 ppm Cr^3+^	3.27	10.35
S80/20c	100 ppm Cr^6+^ and 100 ppm Cr^3+^	4.36	11.42
S50/50c	200 ppm Cr^6+^	8.21	11.41
S50/50c	200 ppm Cr^3+^	3.27	8.80
S50/50c	100 ppm Cr^6+^ and 100 ppm Cr^3+^	4.36	9.53
S20/80c	200 ppm Cr^6+^	8.21	10.26
S20/80c	200 ppm Cr^3+^	3.27	8.83
S20/80c	100 ppm Cr^6+^ and 100 ppm Cr^3+^	4.36	9.18
